# Percutaneous transforaminal endoscopic discectomy in a nine-year-old patient with sciatica: case report, technical note and overview of the literature

**DOI:** 10.1007/s00381-021-05135-6

**Published:** 2021-03-27

**Authors:** P. S. Gadjradj, B. S. Harhangi

**Affiliations:** 1 Department of Neurosurgery, Park MC, Hoofdweg 90, Rotterdam, The Netherlands; 2 Department of Neurosurgery, Erasmus MC University Medical Center Rotterdam, Rotterdam, The Netherlands

**Keywords:** Endoscopic discectomy, Lumbar disk herniation, Pediatrics

## Abstract

Percutaneous transforaminal endoscopic discectomy (PTED) is an alternative procedure to open microdiscectomy (OM) to treat sciatica caused by lumbar disk herniation. Even though robust evidence comparing PTED with OM is lacking, PTED is becoming increasingly popular to treat spinal disorders. In this technical report, the surgical technique and outcomes of PTED in a 9-year-old patient are described. Furthermore, an overview of the literature on full-endoscopic techniques to treat sciatica is given, showing that PTED is feasible, safe and effective to treat lumbar disk herniation in the pediatric population.

## Introduction

In contrary to the adult population in which sciatica caused by lumbar disk herniation is frequently observed, pediatric lumbar disk herniation is a rare entity [1, 2]. Whereas lumbar disk herniation among adults is a result of degeneration of the spine with dehydrated disks, lumbar disk herniations in the pediatric population are typically a result of hydrated disks and are more associated with trauma [3]. Even though sciatica in the pediatric population responds worse to conservative treatment than in the adult population, conservative management remains the first-line treatment of sciatica as it is less invasive for the developing spine [4, 5].

However, when conservative treatment fails, surgery should be considered [6]. Throughout the years, attempts were made to reduce the surgical invasiveness of conventional microdiscectomy by developing other “minimally invasive” techniques [7]. One of these techniques is percutaneous transforaminal endoscopic discectomy (PTED), which is usually associated with less surgical trauma as no paraspinal muscles are detached from their insertion and the bony integrity is largely preserved [8]. There is, however, a paucity in research on the surgical treatment of sciatica in the pediatric population and more specifically in the minimally invasive management.

Therefore, the current study was aimed to describe the PTED-technique with its technical nuances to treat pediatric lumbar disk herniation, and to give an overview of the literature on the use of full-endoscopic techniques to treat lumbar disk herniation in patients aged 18 years or lower.

## Case presentation

**Fig. 1:**
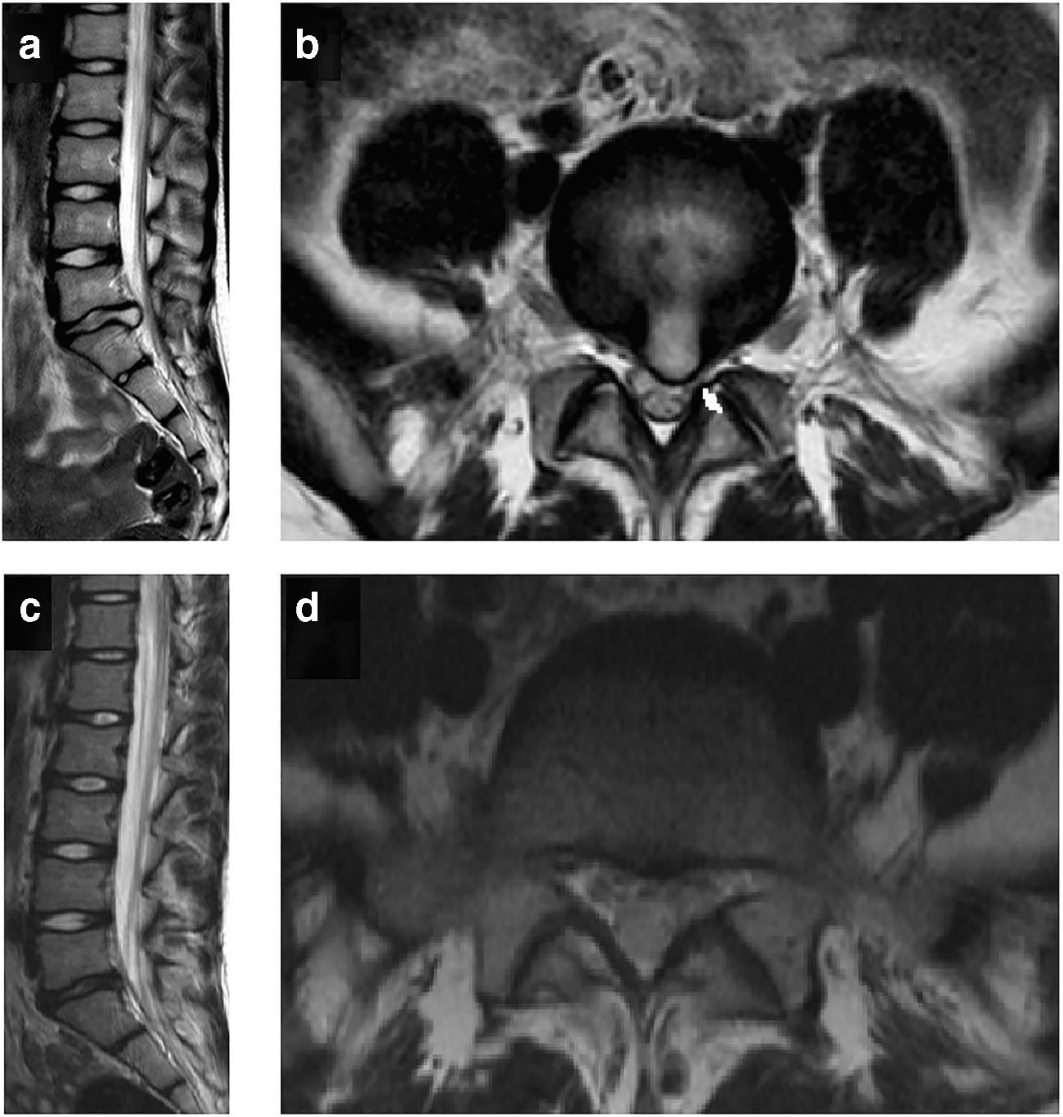
Magnetic resonance imaging showing a median/paramedian lumbar disk herniation at L5-S1 on the left site in sagittal (**a**) and axial plane (**b**). **c**, **d** the sagittal and axial MRI, respectively, six months after surgery. No residual disk herniation is visible and the nerve root at L5-S1 at the left site is adequately decompressed

A 9-year-old female patient had been suffering from sciatica in her left leg for 6 months. Aside from the disabling pain in her left leg, she suffered from dysesthesia. Upon neurological examination, she had a mild paresis distal in her left leg and a positive straight leg raise test at 30 degrees. Apart from her father undergoing lumbar discectomy at 11 years old and her parental grandfather having rheumatoid arthritis, no other diseases have been reported in the family. Magnetic resonance imaging revealed a paramedian disk herniation at L5-S1 on the left side (Fig. 1a, b). She was firstly managed conservatively with physical therapy and pain relieving medications. Because of progressive symptoms, she received an epidural steroid injection which did not show sufficient efficacy. Eventually, she also developed a contracture in her left foot and was referred to the neurosurgery department. Because of the preferences of her parents, she was referred to a neurosurgeon with expertise in endoscopic spine surgery. Preoperative clinical outcomes are shown in Table 1.

**Table 1:** Clinical outcomes at baseline, short-term and long-term follow-up

	Baseline	2 hours postoperative	6 months follow-up	12 months follow-up
VAS leg pain (0–10)^†^	8	0	0	0
VAS back pain (0–10)^†^	5	0	0	0
Functional status (1–5)*	4	-	1	1
Quality of life (1–5)*	4	-	1	1
Days per month not able to do daily activities	>21	-	0	0
Self-perceived recovery of symptoms^‡^	-	-	Fully recovered	Fully recovered
Self-perceived recovery of leg pain^‡^	-	-	Fully recovered	Fully recovered
Satisfied with change^‡^	-	-	Fully satisfied	Fully satisfied
Anxiety for local anesthesia (0–10)^⌠^	7	-	-	-
Satisfaction with local anesthesia (0–10)^⌠^	-	9	-	-

^†^The visual analogue scale for leg pain and back pain measures pain from a 0 to 10 scale with “0” indicating no pain and “10” maximal pain

*The questions regarding functional status and quality of life measures these two outcomes on a five-point scale with “1” indicating an excellent functional status/quality of live and “5” the worst functional status/quality of life

^‡^Self-perceived recovery and satisfaction was measured using a 7-point Likert scale with fully recovered/satisfied and almost fully recovered/satisfied dichotomized as fully recovered/satisfied

^⌠^Anxiety and satisfaction with local anesthesia was measured with a 0 to 10 VAS with “0” indicating no anxiety/fully unsatisfied and “10” maximal anxiety/ fully satisfied

## Surgical technique

The PTED-technique as performed for adults has been extensively described previously [8]. Local anesthesia with lidocaine and intravenous analgesia and sedation are used utilizing a combination of dexmedetomidine and remifentanil. The goal is to have the patient under conscious sedation to enable intraoperative feedback of the patient.

**Fig. 2:**
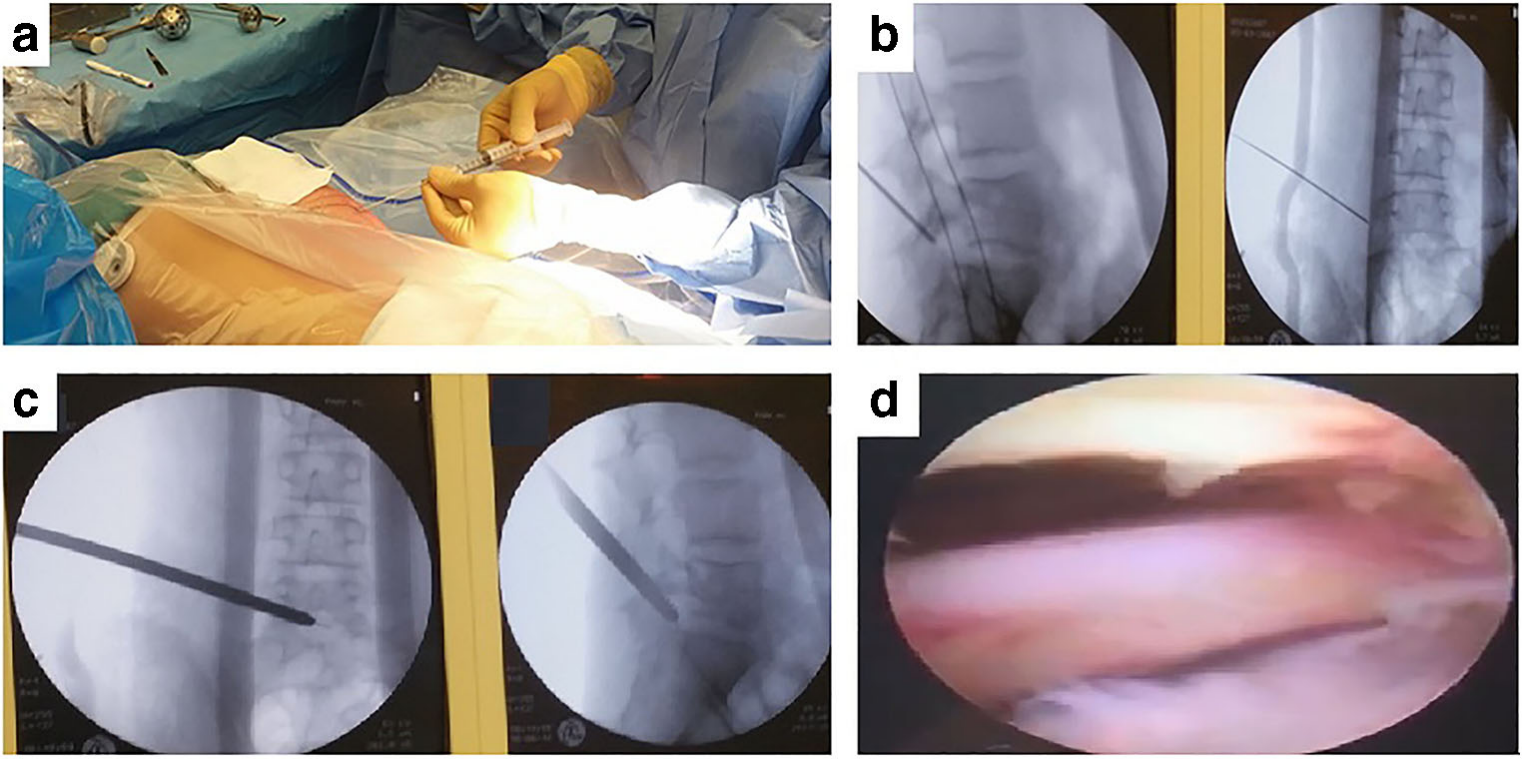
Preoperative images with (**a**) the skin entry point shown at 8-9 cm off the midline, (**b**) the insertion of the guidewire into the neural foramen, (**c**) insertion of the cannula, and (**d**) the view through the endoscope showing the nerve root

The patient was positioned prone on a Wilson frame with the patient’s arms positioned cranially. After disinfection, a sterile screen-drape was applied. Biplane fluoroscopy was used for radiography. The skin entry point was indicated 8–9 cm from the midline superior to the iliac crest with an oblique angle to the L5-S1 disk level (Fig. 2a) which is shorter in comparison to adults which is 12–14 cm from the midline. After injecting the skin entry point with 3 mL of 1% lidocaine, an 18G needle was introduced with its tip located at the superior articular process (SAP) of S1. There, 2 mL of 1% lidocaine was injected, and then, the needle was advanced through the lateral part of the SAP into the spinal canal. Then, a guidewire was inserted through the spinal needle (Fig. 2b). At this stage, an 8-mm skin incision was made, and a standard endoscopic transforaminal procedure was performed with widening of the foramen. Then, an 8-mm working cannula was inserted into the disk space, crossing the medial pedicular line into the disk herniation in the spinal canal (Fig. 2c). The endoscope was introduced through the cannula, and a continuous irrigation system was used to ensure clear endoscopic view, identifying the SAP, posterior longitudinal ligaments, disk herniation, and the spinal canal (Fig. 2d). Decompression was performed with different types of forceps via the working channel of the endoscope until free pulsation of the S1 nerve was identified. After decompression, a single subcutaneous suture and a sterile strip were applied on skin incision. Total duration of surgery was 48 minutes and blood loss was less than 50 mL.

## Discussion

This case report and technical note describe the PTED-technique as performed for a nine-year-old patient. Postoperatively, she experienced no further back or leg pains. These outcomes remained so until the last follow-up time one year after surgery (Table 1). No complications occurred. Even though our patient was anxious about the procedure before surgery, her postoperative experience was satisfactory.

The results of the literature review are shown in Table 2. Five studies reporting the outcomes of full-endoscopic surgery in lumbar disk herniation in children could be identified [9–13]. A total of 110 patients were described undergoing percutaneous endoscopic discectomy either under general or local anesthesia. According to the modified McNab score, 93.6% of the patients (103 out of 110) had an excellent or good outcome after surgery. Furthermore, the complication rates were low and reported only transient sensor or motor symptoms. Two patients developed recurrent disk herniation which required re-discectomy during follow-up.

**Table 2:** Overview of the literature on full-endoscopic procedures to treat sciatica caused by lumbar disk herniation in the pediatric population (age ≤18 years)

Author and year	Sample size	Age (years)	Technique	Anesthesia	Level	Clinical outcomes	Complications/recurrence	Follow-up
Chen et al. 2018 [9]	19	13–18	Transforaminal Interlaminar	Local	L4-5, L5-S1	McNab: excellent (52.6%), good (47.4%)	1× recurrence	41.7 months
Lee et al. 2006 [10]	46	13–18	PELD		L3-4, L4-5, L5-S1	McNab: excellent or good (91.3%).	1× transient dysesthesia 1× recurrence	37.2 months
Mayer et al. 1996 [11]	4	8–17	Transforaminal	Local	L4-5, L5-S1	McNab: excellent (75%), good (25%).	None	1–5 years
Wang et al. 2014 [12]	29	13–18	Interlaminar	General	L4-5, L5-S1	McNab: excellent (83%), good (10%), and fair (7%)	1× transient pareses	19.7 months
Zheng et al. 2016 [13]	12	11–16	Transforaminal	Local	L3-4, L4-5, L5-S1	McNab: excellent (50%), good (41.7%) and fair (8.3%)	1× transient paresthesia	≥ 12 months

Some limitations of the PTED-technique have to be acknowledged. One issue may be the reimbursement of the technique which can oppose a financial barrier to providing PTED. These reimbursement issues may also be caused by the lack of high-level evidence on the merits of PTED over conventional microdiscectomy [14, 15]. Aside from these reimbursement and scientific issues, local anesthesia may also not be suited for all children. Finally, as intraoperative fluoroscopy is extensively used during surgery, children are also exposed to radiation.

In conclusion, PTED is a safe and effective treatment option for pediatric lumbar disk herniation. However, PTED may not be feasible among all pediatric patients as it is usually performed under conscious sedation.

## Abbreviations

OM: open microdiscectomy
PTED: percutaneous transforaminal endoscopic discectomy
RCT: randomized controlled trial
VAS: visual analogue scale
SAP: superior articular process
